# Supplementary material for: Cirscan: a shiny application to identify differentially active sponge mechanisms and visualize circRNA–miRNA–mRNA networks
Source: BMC Bioinformatics. 2024 Feb 1;25:53. doi: 10.1186/s12859-024-05668-y (PMC10832221; doi:10.1186/s12859-024-05668-y)
Supplement: Supplementary file 1 — Additional file 1. Supplementary materials and figures. [file 12859_2024_5668_MOESM1_ESM.pdf]

Supplementary Material

Cirscan: a shiny application to identify differentially active sponge mechanisms and visualize circRNA-miRNA-mRNA networks.

Rose-Marie Fraboulet[1], Yanis Si Ahmed[1], Marc Aubry[2], Sébastien Corre[1], Marie-Dominique Galibert[1,3], and Yuna Blum[1*]

[1]Univ Rennes, CNRS, INSERM, IGDR (Institut de Génétique et Développement de Rennes) - UMR 6290, ERL U1305, F-35000 Rennes, France.
[2]INSERM, OSS (Oncogenesis Stress Signaling), UMR_S 1242, CLCC Eugene Marquis, Univ Rennes 1, 35000, Rennes, France.
[3]Department of Molecular Genetics and Genomics, Hospital University of Rennes (CHU Rennes), F-35000 Rennes, France.

Contents

1 Cirscan Description **1**
1.1 In-house database of miRNA-target interactions based on predicted and experimentally validated interactions . . . 1

2 Cirscan performace evaluation **3**
2.1 Pre-processing of colorectal cancer data . . . 3
2.2 Pre-processing of hepatocarcinoma data . . . 3
2.3 TargetScan performance evaluation . . . 3

3 Supplemental References **5**

1 Cirscan Description

1.1 In-house database of miRNA-target interactions based on predicted and experimentally validated interactions

A miRNA-target interaction is established between a region of the miRNA, called "seed sequence" and a complementarity sequence called "miRNA Recognition Element" (MRE) on the target (circRNA or mRNA). An in-house database of interactions between miRNAs and putative mRNA or circRNA targets was constructed using the TargetScan interaction prediction tool (McGeary et al. 2019) and information from experimentally validated interaction from the ENCORI database (Li et al. 2014).

TargetScan was used to predict miRNA-circRNA and miRNA-mRNA interactions, and to obtain an affinity score for each interaction, by taking into account the specificity of the RNA type considered (mRNA or circRNA). For miRNA-circRNA interactions, we used 140,790 circRNA sequences from circBase (Glažar, Papavasileiou, and Rajewsky 2014) and 9,994 miRNA sequences from TargetScan (Garcia et al. 2011). TargetScan v6 calculates for each miRNA-circRNA interaction an affinity score called context+ score, corresponding to the sum of the contribution of six features (site-type, 3'-compensatory pairing, local AU, position, target site abundance and seed-pairing stability). The feature 3'-compensatory pairing was removed here because it is not sufficiently justifiable at present,

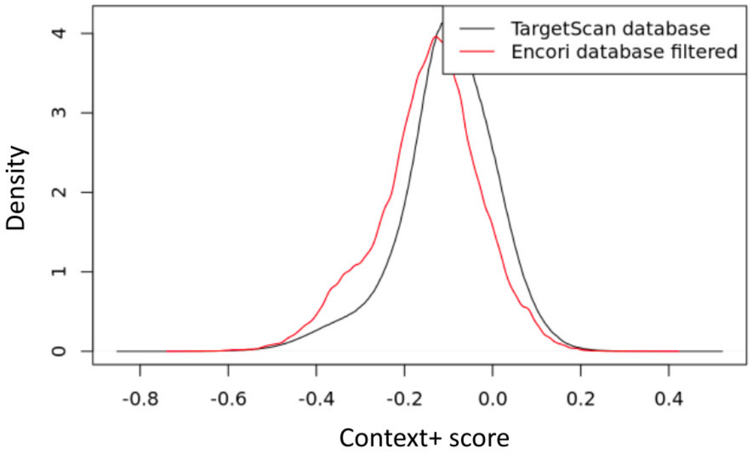

Figure S1: Context+ score distribution on miRNA-circRNA interactions predicted by the TargetScan database and on experimentally validated miRNA-circRNA interactions (CLIP-seq and degradome-seq >= 2) by the ENCORI database.

as the specific mechanisms of circRNA-miRNA pairing remain poorly described in the literature. The lower the context+ score, the stronger the affinity between the miRNA and its target. To reduce the number of false positive predictions, we defined a cutoff on the context+ score, based on its distribution on experimentally validated interactions given by the ENCORI database. We defined as a cutoff the 95th percentile of the context+ score distribution restricted to the interactions supported by at least two CLIP-seq and two degradome-seq experiments (Figure S1). We used the ENCORI/Starbase API [`http://starbase.sysu.edu.cn/tutorialAPI.php`] to download the experimentally validated interactions contained in this database.

We retrieved 2,055,407 miRNA-circRNA experimentally validated interactions:

```
curl 'https://starbase.sysu.edu.cn/api/miRNATarget/?assembly=hg19&
    geneType=circRNA&miRNA=all&clipExpNum=0&degraExpNum=0&pancancerNum=0&
    programNum=1&program=None&target=all&cellType=all'
```

and 1,215,274 miRNA-mRNA experimentally validated interactions:

```
curl 'https://starbase.sysu.edu.cn/api/miRNATarget/?assembly=hg19&
    geneType=mRNA&miRNA=all&clipExpNum=0&degraExpNum=0&pancancerNum=0&
    programNum=1&program=None&target=all&cellType=all'
```

For miRNA-mRNA interactions, we used 2,382,569 UTR sequences and 9,994 miRNA sequences from TargetScan (Garcia et al. 2011). TargetScan v8 calculates for each miRNA-mRNA interaction an affinity score called context++ score (Agarwal et al. 2015). Compared to the context+ score, the context++ score includes additional criteria specifically relevant for miRNA-mRNA interactions (e.g. 3' UTR length, ORF length, probability of conserved targeting between species), which are expected to reduce false positive predictions.

MiRNA-circRNA and miRNA-mRNA interactions were restricted to specific MRE binding sites: 7mer-m8 (exact match to positions 2-8 of the mature miRNA), 7mer-1a (exact match to positions 2-7

of the mature miRNA followed by an "A"), and 8mer-1a (exact match to positions 2-8 of the mature miRNA followed by an "A"), i.e. 1,244,932 miRNA-mRNA interactions and 31,045,182 miRNA-circRNA interactions in total.

2 Cirscan performace evaluation

2.1 Pre-processing of colorectal cancer data

Microarray multi-level transcript expression data of 10 colorectal cancer (CRC) samples and 10 normal adjacent samples were downloaded from the NCBI Gene Expression Omnibus database (accession number: GSE126095). Each dataset was imported into the RStudio (v1.4.1103) environment with R (v4.2.2) for pre-processing.

For transcripts belonging to the gene annotation, an expression average was applied. Quantile normalization and a log-transformation were applied to the mRNAs expression matrix. mRNAs microarray matrix was reduced to protein-coding genes, using the annotation file provided by the authors. Using the limma R package (v.3.50.1) (Ritchie et al. 2015), 4,640 differentially expressed mRNAs with an adjusted p-value (Benjamini-Hochberg, BH) < 0.05 were selected. The circRNAs microarray matrix was also submitted to quantile normalization and a log-transformation. We selected 1,491 differentially expressed circRNAs with an adjusted p-value (BH) < 0.05 by using the limma R package (v.3.50.1) (Ritchie et al. 2015). Finally, the miRNAs microarray matrix was filtered to only include miRNA identifiers present in the annotation file, i.e. 2,055 miRNAs. Quantile normalization and log-transformation were also applied to the miRNAs expression matrix. These different pre-processed expression matrices were given as input to Cirscan.

2.2 Pre-processing of hepatocarcinoma data

circRNAs microarray expression data of 7 hepatocellular carcinoma (HCC) tissues and 7 non-tumor liver tissues were downloaded from the NCBI Gene Expression Omnibus database (accession number: GSE97332). mRNAs expression matrix of 424 liver samples (374 tumor tissues and 50 normal tissues) were downloaded from the TCGA (`https://www.cancer.gov/tcga`). Each dataset was imported into the RStudio (v1.4.1103) environment with R (v4.2.2) for pre-processing.

circRNAs identifiers have been converted according to the nomenclature described by circBase (i.e. 3,416 unique circRNAs). The circRNAs microarray expression matrix was restricted to 2,222 differentially expressed circRNAs with an adjusted p-value (BH) < 0.05 by using the limma R package (v.3.50.1) (Ritchie et al. 2015). mRNAs counts expression matrix was reduced to protein-coding genes, using the annotation file provided by using the Ensembl database and the Biomart web tool (Howe et al. 2021, `https://www.ensembl.org/index.html`). As mRNA expression data was already log-transformed, we performed a conversion to raw data ($2\wedge$x-1 transformation) in order to use them with the DESeq2 R package (v1.40.2) (Love, Huber, and Anders 2014), and we selected 10,760 mRNAs differentially expressed with an adjusted p-value (BH) < 0.05. These different pre-processed expression matrices were given as input to Cirscan. Finally, the miRNAs expression data was directly retrieved from the signature of sufficiently expressed miRNA from liver cancer available in the Cirscan tool, as no miRNA expression dataset was generated by the same authors.

2.3 TargetScan performance evaluation

To demonstrate the efficiency of TargetScan predictions over random predictions in the context of sponge mechanisms, we constructed random miRNA-target prediction databases. This involves permuting the values of criteria related to TargetScan in databases, such as $S_{Affinity}$, S_{NbMRE} and $S_{EnrichMRE}$ for the circRNA-miRNA interaction database and $S_{Affinity}$, S_{NbMRE} for the mRNA-miRNA database. We performed 500 permutations and applied Cirscan to our two benchmark datasets

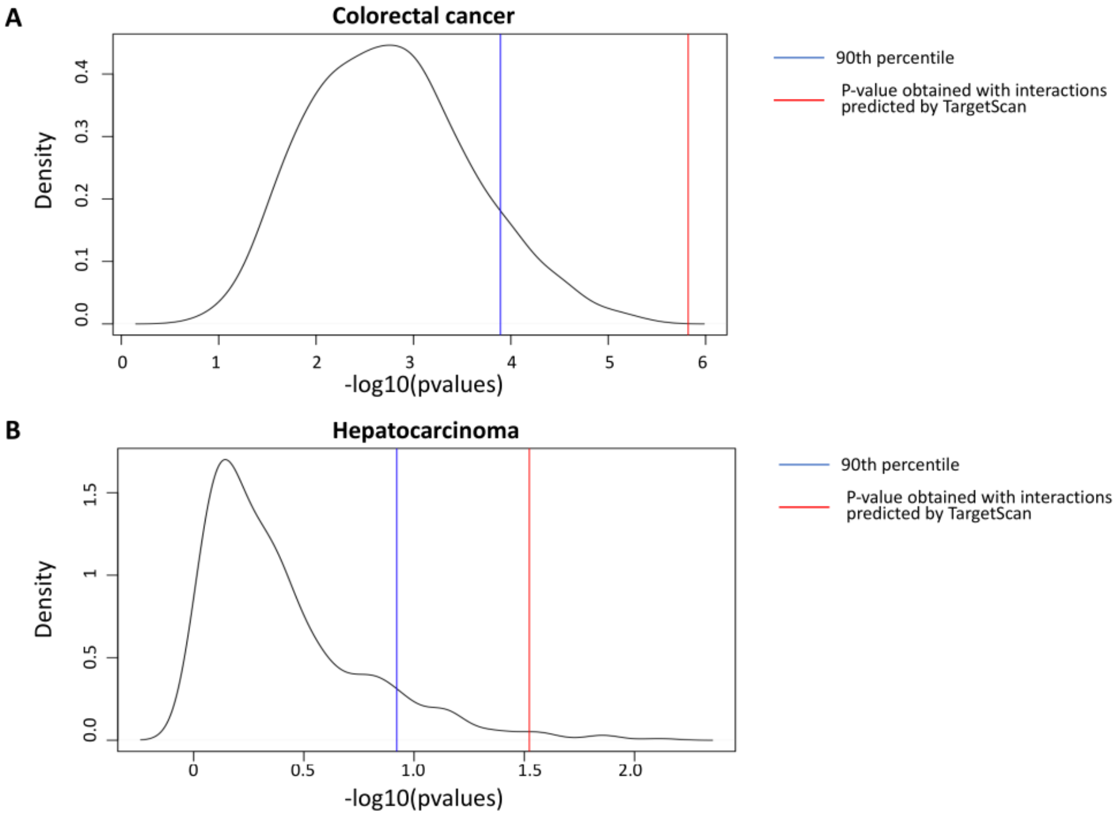

Figure S2: Distribution of the -log10 p-values of the enrichment analysis of the validated sponge mechanism (500 permutations on TargetScan miRNA-target interaction databases) for A) the colorectal benchmark dataset, B) hepatocarcinoma benchmark dataset. The blue line corresponds to the 90th percentile of this distribution, and the red line to the enrichment p-value obtained with databases of interactions predicted by TargetScan (as described in the main text).

(public colorectal cancer and hepatocarcinoma data). Our analysis focused on the enrichment of published mechanisms for these two cancers among the top-ranked sponge mechanisms identified by Cirscan. To assess statistical significance, we compared the distribution of enrichment p-values (-log10) of the 500 permutation results with the one obtained using TargetScan (described in the main text). As shown in Figure S2, the enrichment p-value (-log10) obtained with TargetScan predictions is greater than the 90th percentile of the distribution of 500 p-values (-log10) obtained with random prediction databases for both cancers. It is important to note that the sponge score calculated by Cirscan takes into account multiple criteria, some based on Targetscan predictions but also others based on expression levels. The final score is therefore a balance between all these criteria, which can explain significant p-values of the benchmark analysis using randomly generated target-miRNA pairs (Figure S2). However, it is noteworthy that these p-values remain notably less significant than those obtained using the true Targetscan predictions. Overall, this analysis shows the relevance of the predictions identified by TargetScan and the importance of this criteria in the identification of sponge mechanisms identified by Cirscan.

3 Supplemental References

Agarwal, Vikram et al. (Aug. 12, 2015). "Predicting effective microRNA target sites in mammalian mRNAs". In: *eLife* 4, e05005. ISSN: 2050-084X. DOI: 10.7554/eLife.05005. URL: https://elifesciences.org/articles/05005 (visited on 11/29/2021).

Garcia, David M. et al. (Oct. 2011). "Weak seed-pairing stability and high target-site abundance decrease the proficiency of lsy-6 and other microRNAs". In: *Nature Structural & Molecular Biology* 18.10. Number: 10 Publisher: Nature Publishing Group, pp. 1139–1146. ISSN: 1545-9985. DOI: 10.1038/nsmb.2115. URL: https://www.nature.com/articles/nsmb.2115 (visited on 03/16/2022).

Glažar, Petar, Panagiotis Papavasileiou, and Nikolaus Rajewsky (Sept. 18, 2014). "circBase: a database for circular RNAs". In: *RNA*. Company: Cold Spring Harbor Laboratory Press Distributor: Cold Spring Harbor Laboratory Press Institution: Cold Spring Harbor Laboratory Press Label: Cold Spring Harbor Laboratory Press Publisher: Cold Spring Harbor Lab. ISSN: 1355-8382, 1469-9001. DOI: 10.1261/rna.043687.113. URL: http://rnajournal.cshlp.org/content/early/2014/09/18/rna.043687.113 (visited on 04/19/2021).

Howe, Kevin L. et al. (Jan. 8, 2021). "Ensembl 2021". In: *Nucleic Acids Research* 49 (D1), pp. D884–D891. ISSN: 1362-4962. DOI: 10.1093/nar/gkaa942.

Li, Jun-Hao et al. (Jan. 2014). "starBase v2.0: decoding miRNA-ceRNA, miRNA-ncRNA and protein-RNA interaction networks from large-scale CLIP-Seq data". In: *Nucleic Acids Research* 42 (Database issue), pp. D92–97. ISSN: 1362-4962. DOI: 10.1093/nar/gkt1248.

Love, Michael I., Wolfgang Huber, and Simon Anders (Dec. 5, 2014). "Moderated estimation of fold change and dispersion for RNA-seq data with DESeq2". In: *Genome Biology* 15.12, p. 550. ISSN: 1474-760X. DOI: 10.1186/s13059-014-0550-8. URL: https://doi.org/10.1186/s13059-014-0550-8 (visited on 09/25/2023).

McGeary, Sean E. et al. (Dec. 20, 2019). "The biochemical basis of microRNA targeting efficacy". In: *Science* 366.6472, eaav1741. ISSN: 0036-8075, 1095-9203. DOI: 10.1126/science.aav1741. URL: https://www.sciencemag.org/lookup/doi/10.1126/science.aav1741 (visited on 03/17/2021).

Ritchie, Matthew E. et al. (Apr. 20, 2015). "limma powers differential expression analyses for RNA-sequencing and microarray studies". In: *Nucleic Acids Research* 43.7, e47. ISSN: 1362-4962. DOI: 10.1093/nar/gkv007.
